# How and Who? Examining Real‐World Evidence of Engagement and Use of an Electronic Patient‐Reported Outcome Smartphone Application in Routine Clinical Care for Patients With Inflammatory Arthritides

**DOI:** 10.1002/acr2.70049

**Published:** 2025-05-06

**Authors:** Iuliia Biliavska, Erik Lenguerrand, Jonathan H. Tobias, Philip D. H. Hamann

**Affiliations:** ^1^ University of Bristol, Bristol, United Kingdom, and State Institution, National Scientific Center, The M.D. Strazhesko Institute of Cardiology, Clinical and Regenerative Medicine of the National Academy of Medical Sciences of Ukraine Kyiv Ukraine; ^2^ University of Bristol Bristol United Kingdom; ^3^ North Bristol NHS Trust and Bristol Medical School University of Bristol Bristol United Kingdom

## Abstract

**Objective:**

We evaluate the use of an app to remotely collect electronic patient‐reported outcomes (ePROs) in patients attending routine rheumatology clinics with inflammatory arthritis (IA) over a four year period.

**Methods:**

This is a secondary analysis of real‐world data obtained from patients with IA who attended routine appointments between 2018 and 2022. Patients used an app to track their disease course by using Routine Assessment of Patient Index Data 3 (RAPID3), Health Assessment Questionnaire ‐ Disability Index, or self‐assessing tender and swollen joint counts. Elapsed days of the app use, number, and time between ePROs reports were analyzed using Poisson and Tobit regression models. Results were stratified by gender, age, diagnosis, baseline disease severity, and disability.

**Results:**

At least one ePRO report was provided by 673 patients. Mean age was 53.7 ± 13.9 years; 458 (68%) were female. RAPID3 was reported by 613 (91%) patients, 531 (79%) provided more than one RAPID3, and 82 (12%) provided one RAPID3; there was no difference between groups stratified by gender, age, IA type, and baseline arthritis severity. Median engagement was 14.8 months (interquartile range 12.8–17). The proportion of enrolled patients completing a RAPID3 every month reduced from 91% at baseline to 38% at 6 months, and 24% at month 12. Older patients (60–69 years old) were more likely to be long‐term users than those aged less than 50 years old (*P* < 0.006). Patients aged over 60 provided more reports than younger users (*P* < 0.0001). Gender, baseline arthritis activity, and disability level were not associated with the length or frequency of app use.

**Conclusion:**

This analysis offers insights into engagement and long‐term sustainability of remote ePRO collection in a real‐world rheumatology setting.

## INTRODUCTION

Inflammatory arthritis (IA), including conditions such as rheumatoid arthritis (RA), axial spondylarthritis, psoriatic, and undifferentiated arthritis,^1^, ^2^ can cause stiffness, pain, swelling, and functional decline in the affected joints that fluctuates over time.^3^ Because of the chronic and unpredictable nature of IA, it can be difficult to identify the optimal timing for a patient's clinical review. Patients who experience disease flares between visits may forget to inform treating clinicians, leading to false reassurance at clinical reviews and suboptimal management. Difficulty quantifying and tracking disease activity can make it challenging for patients to frame current IA severity in the context of their arthritis course and can lead to patients feeling that the disease controls them and not the reverse.^4^

**SIGNIFICANCE & INNOVATIONS**

- User‐level engagement is markedly different between research studies and real‐world rheumatology clinical practice. Outside of research settings, engaging patients to use an electronic patient‐reported outcomes (ePRO) smartphone application (app) for more than six months is challenging.
- Older participants (aged 60–69) are more engaged users of such apps, using them longer and providing more reports. Different strategies may be needed to engage and retain younger patients with inflammatory arthritis to use digital health tools for remote ePRO collection.
- The long‐term engagement use of participants is independent of gender, baseline disease severity, and disability level.

Patient‐reported outcomes (PROs) are important indicators of health and well‐being that convey true disease experience and impact of symptoms, functional limitations, and treatment effectiveness.^5^, ^6^ They are reported directly by the patient without clinician input, and validated scores allow clinicians to understand the disease activity from the patient's perspective.^7^ Technological innovations and increased use of electronic devices have facilitated the widespread adoption of electronic PROs (ePROs).^8^ The benefits of integrating regular remotely reported ePROs within clinical pathways are well documented and have contributed to a paradigm shift from disease‐centered to patient‐centered care.^9^, ^10^, ^11^, ^12^ ePROs have an increasing role in creating individualized treatment plans and enable better tracking of outcome changes, and with the advent of accessible artificial intelligence, longitudinal ePRO data could be used to develop algorithms that predict future disease activity.^13^, ^14^

NHS (NHS) England has highlighted an urgent need to adopt digital tools across the health care system to address long‐term improvement goals, with ambitions for health and social care services to have digital foundations in place by 2025.^15^ However, widespread uptake of ePROs in routine clinical practice remains limited. Their implementation in rheumatology has mostly occurred in the context of clinical trials with a defined duration.^11^, ^12^, ^16^, ^17^, ^18^ In contrast, clinical practice requires long‐term, often lifelong, care. Insufficient patient engagement and retention are two widely acknowledged barriers to interpreting and translating findings from digital health research.^19^ Promising retention rates seen in research trials are unlikely to be replicated in routine care and real‐world rheumatology practice involves new challenges, including lack of access to digital tools as well as variable health and technology literacy among patients.^20^

Our understanding of factors that impact patient retention rate and real‐world evidence describing long‐term engagement (beyond 12 months) with digital tools remains limited.^21^ Such knowledge is essential for the successful implementation and sustainable use of remote monitoring at scale in routine rheumatology practice. Health care providers need to know when, how many, and which patients are likely to engage for long enough to make changes in clinical care worthwhile. Knowing which patients are less likely to engage will also help stakeholders focus their efforts on a better understanding of engagement barriers and build solutions to overcome them. This analysis aims to understand the pattern and examine determinants of long‐term engagement, beyond four years, with a self‐reporting ePRO smartphone application (app) in the management of patients with IA as part of usual clinical care.

## PATIENTS AND METHODS

### Patient population

This study is a secondary analysis of data obtained in a real‐world clinical setting from patients diagnosed with IA (including RA, psoriatic arthritis, spondyloarthritis, or undifferentiated arthritis).^22^ Patients aged ≥18 years old, with a clinical diagnosis of IA, and who attended routine appointments in the rheumatology outpatient clinic at North Bristol NHS Trust between 2018 and 2022 were offered access to an app (Living With). The app was used to record ePROs between appointments including Routine Assessment of Patient Index 3 (RAPID3),^23^ Health Assessment Questionnaire ‐ Disability Index (HAQ‐DI),^24^ and self‐assessed tender or swollen joint counts.^25^ Health care professionals provided patients with initial instructions and an introduction to the app. Patients were advised to report disease severity (using RAPID3) every week and physical function (HAQ‐DI) every 28 days. Data were analyzed from patients with at least one record until the last registered follow‐up. Disease severity and disability categories were defined using validated thresholds for baseline RAPID3 and HAQ‐DI scores.^24^, ^26^ Data were not used to alter access to rheumatology services. Patients were informed that their data would be used to enrich and facilitate history taking and would only be reviewed during consultations unless the clinical team was specifically asked to review them between appointments.

### Technology description

Patients were required to have a smartphone or tablet running Android or iOS and email address to use the app. The invitation email included the download instructions. Patients were advised to inform their clinician during the consultation if they were using the app. Clinicians then viewed the data during the consultation via the web‐based clinical portal and data were used to enrich and facilitate history taking in consultations. No formal data were collected regarding clinician access or use of the data in clinics. The app included opt‐in pop‐up reminders if a patient did not complete ePROs. Patients were able to review their outcome scores in the app. The app was designed with the involvement of patients in co‐design and production and is compliant with the NHS Data Security and Protection Toolkit.^27^ It adheres to the General Data Protection Regulations, and institutional information governance approval was granted. The ePROs were collected over 48 months, stored locally on the smartphone, and transferred to a secure cloud database with restricted access via a web‐based portal. Only authorized clinicians were able to access the portal. Clinicians could view patient‐level data in graphical and tabular format (Supplementary Figure 1). All analyzed data were anonymized.

### Statistical analysis

Categorical variables were presented as frequencies and percentages. Continuous variables that were not normally distributed were presented as median (25th and 75th percentile interquartile range, IQR).

Patients who provided more than one ePRO were classified as *follow‐up users*. Those who only reported baseline ePRO were identified as *lost to follow‐up users*.

Patient engagement (app survival) was reported using a Kaplan‐Meier curve.^19^ We considered an *engaged* user as someone who completed at least one ePRO up until 12 weeks before data cut‐off in July 2022, to allow all patients included in the analysis an equal, minimum period to engage. The retention rate was defined as the proportion of patients submitting a RAPID3 each month among patients who had been registered with the app up until that point.^28^

The difference between the baseline report and the last recorded ePRO entry was calculated as maximum elapsed days. The variables ‘number of ePROs’ and ‘days between ePROs’ (average interval in days between ePROs per patient) were also analyzed. In the case of multiple reports obtained from the patient in one day, the mean value was calculated. A Poisson regression model was used to assess the difference between follow‐up and lost to follow‐up app‐user subpopulations by demographics, diagnosis, and disease characteristics.

In the follow‐up user group, Tobit regression modeling was used to assess the association between the patient demographic or disease‐related parameters and outcomes (elapsed days, number of ePROs, and days between ePROs).^29^ Results were stratified by gender, age, diagnosis, and baseline disease activity. Statistical tests were conducted using Stata 16.1 (StataCorp) and RStudio (version 1.4.1717). A *P* value of < 0.05 was used to define associations that were unlikely to have occurred by chance. The analysis was approved by Health Research Authority (HRA) and Health and Care Research Wales (23/HRA/2142).

## RESULTS

**Figure 1:**
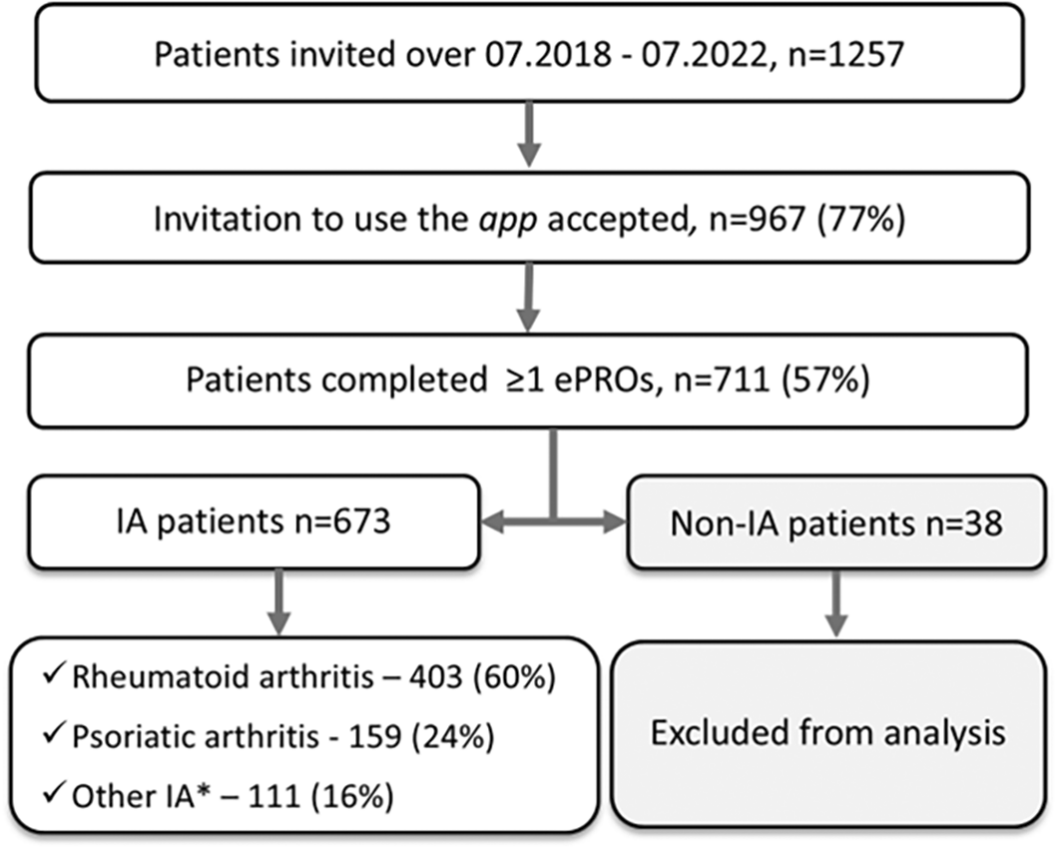
Study flow chart of population selection. *Other IA: undifferentiated arthritis, 48 (7.1%); axial spondyloarthritis, 28 (2.9%); enteropathic arthritis, 13 (1.9%); juvenile idiopathic arthritis, 12 (1.8%); reactive arthritis, 10 (1.5%). ePRO, electronic patient‐reported outcome; IA: inflammatory arthritis.

A total of 1,257 patients were invited to use the app. Of these, 967 (77%) downloaded the app. A total of 711 (57%) patients provided at least one ePRO, of which 38 (5%) were excluded due to non‐IA diagnosis. Data from the remaining 673 patients with IA were included for further analysis (Figure 1).

The rate of app downloads rose notably in April 2020, peaking in mid‐May and mid‐June 2020, corresponding with the initial phase of the COVID‐19 pandemic. The first completion of any ePROs occurred a median of one day after registration (IQR 0–15 days; Supplementary Figure 2A, B).

Of the 673 patients with IA who completed any of the baseline ePROs, 458 (68%) were female. The mean user age was 53.7 ± 13.9 years. Most participants had RA (403; 60%), followed by psoriatic arthritis (159; 24%), and the remaining (111; 16%) had other types of IA. RAPID3 severity score was most commonly completed (91%), followed by self‐assessment of tender or swollen joint counts (89%), and functional disability using HAQ‐DI score (78%). All three available ePROs were reported by 72% of patients. Most patients had moderate to high IA severity according to baseline RAPID3 scores and mild or moderate levels of baseline functional disability considering HAQ‐DI score (Table 1).

**Table 1:** Demographic characteristics of 673 patients with IA*

Demographics	n (%)
Gender	
Woman	458 (68)
Man	215 (32)
Age, mean ± SD, y	53.7 ± 13.9
Diagnosis	
Rheumatoid arthritis	403 (60)
Psoriatic arthritis	159 (24)
Other IA	111 (16)
≥1 report(s) provided	
RAPID3	613 (91)
Joints self‐assessment	602 (89)
HAQ‐DI	527 (78)
Baseline disease severity, RAPID3	
Remission (<3.0)	83 (12)
Low (3.1–6.0)	85 (13)
Moderate (6.1–12.0)	151 (22)
High (>12.0)	294 (44)
Missing data	60 (9)
Baseline functional disability, HAQ‐DI	
Mild (≤1.0)	287 (43)
Moderate (>1.0–≤2.0)	191 (28)
Severe (>2.0–≤3.0)	49 (7)
Missing data	146 (22)

* HAQ‐DI, Health Assessment Questionnaire ‐ Disability Index; IA, inflammatory arthritis; RAPID3, Routine Assessment of Patient Index Data 3.

Of the 613 participants who completed the RAPID3 questionnaire at baseline, 531 (79%) also reported at least one further score and were identified as follow‐up app users. A total of 82 (12%) patients who only provided a baseline report were classified as lost to follow‐up. There was no difference identified in the adjusted Poisson regression model between lost to follow‐up and follow‐up app users by gender, age, IA type, and baseline arthritis severity (Table 2).

**Table 2:** Comparison of patient demographics and disease characteristics in patients who submitted more than one RAPID3 (follow‐up patients) compared with those who submitted only one RAPID3 (lost to follow‐up) via the app*

Characteristics	n (%)	Adjusted^a^ RR (95% CI)	*P* value
Gender			
Woman	359 (86.1)	Ref	–
Man	172 (87.8)	1.03 (0.96–1.10)	0.42
Age, y			
<50	200 (85.1)	Ref	–
50–59	138 (89.6)	1.05 (0.97–1.13)	0.21
60–69	111 (87.4)	1.02 (0.93–1.11)	0.69
>70	82 (84.5)	0.98 (0.88–1.09)	0.70
Diagnosis			
RA	321 (87.5)	Ref	–
PsA	125 (85.6)	0.97 (0.90–1.05)	0.46
Other	85 (85.0)	0.97 (0.88–1.06)	0.47
Disease severity, RAPID3			
Remission (<3.0)	73 (86.9)	Ref	–
Low (3.1–6.0)	70 (85.4)	0.98 (0.87–1.11)	0.80
Moderate (6.1–12.0)	132 (86.3)	1.00 (0.91–1.11)	0.92
High (>12.0)	256 (87.1)	1.01 (0.92–1.11)	0.81

* Follow‐up outcomes: follow‐up, n = 531 versus lost to follow‐up, n = 82. CI, confidence interval; PsA, psoriatic arthritis; RA, rheumatoid arthritis; RAPID3, Routine Assessment of Patient Index Data 3; Ref, reference; RR, risk ratio.

^a^ Poisson regression adjusted for gender, age, diagnosis, and disease severity.

**Figure 2:**
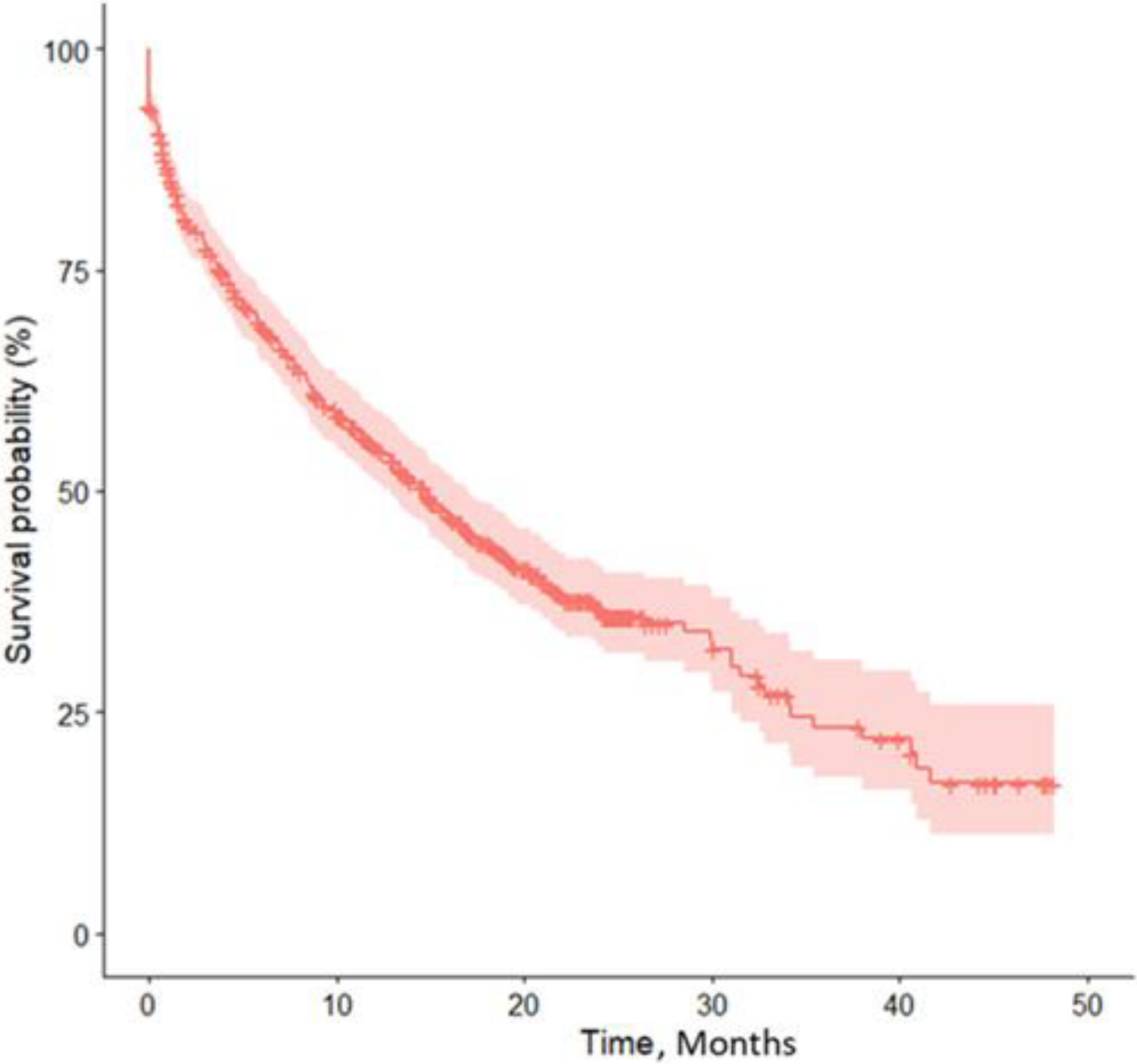
Kaplan‐Meier curve (95% confidence interval) of long‐term patient engagement with RAPID3 score. RAPID3, Routine Assessment of Patient Index Data 3.

Patient engagement with the app decreased over time. Kaplan‐Meier analysis of RAPID3 completion showed that 50% of patients had dropped out by 14.8 months (95% confidence interval 12.8–17 months; Figure 2).

**Figure 3:**
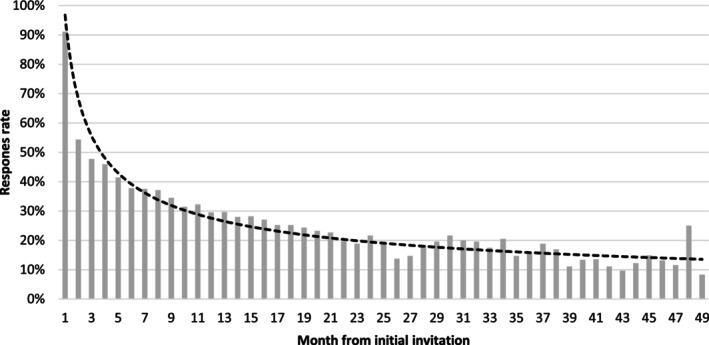
Proportion of registered patients submitting a RAPID3 each month (n = 613).* Patients were recruited from July 2018 to July 2022. The length of follow‐up varies, with patients recruited in 2018 having up to 49 months of follow‐up, and those recruited in July 2022 having up to one month of follow‐up. RAPID3, Routine Assessment of Patient Index Data 3.

The proportion of enrolled patients submitting a RAPID3 each month reduced most in the first six months, from 91% at baseline to 38% at month 6, and then to 24% at month 12. The proportion of patients submitting a RAPID3 each month plateaued thereafter (Figure 3).

Among follow‐up users, older patients (aged 60–69 years old) were more likely to be long‐term users compared with younger participants (≤50 years old), with a median maximum elapsed days of app use of 379 days (IQR 169–645 days) compared with 256 days (IQR 90–515 days; *P* < 0.006). Patients aged 60 to 69 and ≥70 years old provided more RAPID3 reports per patient (median 18; IQR 7–46; and median 25; IQR 8–62, respectively) compared with younger app users (median 8; IQR 4–18; *P* < 0.0001; Table 3). However, there was no difference in frequency of app use as reflected by days between RAPID3 reports comparing patients aged 60 to 69 and older than 70 years (median 7 days; IQR 7–14; and median 7 days; IQR 7–15, respectively) to the younger app users (median 9 days; IQR 7–18; *P* < 0.17).

**Table 3:** Tobit regression model of app use sustainability in the follow‐up user group*

Characteristics		Махimum еlapsed days^a^		Number of ePROs		Days between ePROs	
	n (%)	Median (IQR)	Adjusted^b^ *P* value	Median (IQR)	Adjusted^b^ *P* value	Median (IQR)	Adjusted^b^ *P* value
Gender							
Woman	359 (58.6)	306 (109–565)	Ref	10 (5–30)	Ref	10 (7–18)	Ref
Man	172 (28.1)	359 (133–608)	0.9	16 (6–43)	0.324	7 (7–15)	0.785
Age, y							
<50	200 (32.6)	256 (90–515)	Ref	8 (4–18)	Ref	11 (7–21)	Ref
50–59	138 (22.5)	308 (105–567)	0.256	12 (5–30)	0.253	9 (7–16)	0.86
60–69	111 (18.1)	379 (169–645)	0.006	18 (7–46)	0.00001	7 (7–14)	0.15
>70	82 (13.4)	401 (168–609)	0.06	25 (8–62)	0.00001	7 (7–15)	0.12
*P* value	–	0.03	–	0.0001	–	0.17	–
Severity, RAPID3							
Remission (<3.0)	73 (11.9)	391 (113–609)	Ref	14 (5–47)	Ref	7 (7–14)	Ref
Low (3.1–6.0)	70 (11.4)	337 (133–651)	0.82	15 (5–53)	0.79	8 (7–16)	0.86
Moderate (6.1–12.0)	132 (21.5)	354 (135–585)	0.87	11 (5–30)	0.23	11 (7–18)	0.68
High (>12.0)	256 (41.8)	300 (112–540)	0.27	12 (5–31)	0.09	8 (7–17)	0.83
*P* value	–	0.25	–	0.1	–	0.9	–
Disability, HAQ‐DI							
Mild (≤1.0)	249 (40.6)	371 (119–580)	Ref	12 (5–35)	Ref	9 (7–15)	Ref
Moderate (>1.0–≤2.0)	166 (27.1)	335 (148–588)	0.78	14 (6–39)	0.66	8 (7–18)	0.14
Severe (>2.0–≤3.0)	38 (6.2)	238 (105–588)	0.68	12 (8–20)	0.64	8 (7–17)	0.88
*P* value	–	0.67	–	0.43	–	0.41	–

* n = 531 for the follow‐up user group. ePROs, electronic patient‐reported outcomes; HAQ‐DI, Health Assessment Questionnaire ‐ Disability Index; IQR, interquartile range; RAPID3, Routine Assessment of Patient Index Data 3.

^a^ Махimum elapsed days are defined as the period between baseline report and the last recorded ePRO entry.

^b^ Tobit regression adjusted for gender, age, and disease severity.

No difference in maximum elapsed days of ePRO app use was found according to patients’ gender, baseline RAPID3 disease severity, and HAQ‐DI disability level. The number of RAPID3 reports and days between them were similar when stratified by gender, baseline arthritis severity, and functional disability.

## DISCUSSION

To our knowledge, this is one of the longest real‐world follow‐up studies of app usage of this nature. Our results provide unique insights into how remote monitoring using ePROs evolves in real‐world rheumatology practice and identifies notable factors and determinants of its long‐term usage.

Our data highlight the challenges of implementing digital tools for remote monitoring in routine care. We have shown there is a notable reduction in engagement and usage of the app in the first six months. However, over time, a stable plateau was achieved. This likely reflects patients’ and clinicians’ behavior settling around a usage pattern that fits with their clinical needs along with the loss of patient users in which the initial novelty of the solution has passed. This is in keeping with other studies that have found common barriers such as inadequate engagement and responder fatigue.^30^

In a systematic review, Doumen et al highlighted a high heterogeneity in engagement across digital research studies along with significant differences between digital tools and frequency of PRO collection.^31^ Importantly, this review highlighted the limited availability of data from daily clinical practice. This evidence gap is important because it is well documented that clinical research studies obtain higher levels of engagement than are achieved when innovations are adopted into real‐world settings.^10^ Understanding engagement patterns is particularly important because patient completion of ePROs is essential to the function of most remote monitoring clinical service models in rheumatology.

However, analysis of engagement and persistence of use of remote monitoring tools outside of the clinical trial settings offers additional challenges owing to the lack of standard agreement about what defines ongoing use (such as a minimum reporting frequency), and when a patient is deemed to have dropped out. Furthermore, terms such as *engagement* and *retention rate* are often used interchangeably.^25^

Standard Kaplan‐Meier analysis is commonly used in studies, including ours, as it is widely understood and gives a clear graphical representation of overall trends. However, such analysis was designed for irreversible endpoints, such as death or study withdrawal.^32^ However, when considering long‐term real‐world studies such as ours, patients may not complete ePROs for many months before re‐engaging and completing regular ePROs again, often after a clinic appointment. These patients may be defined as dropped out even though they may subsequently re‐engage. This sporadic engagement is difficult to quantify using Kaplan‐Meier analysis or other standard survival analysis. To enable Kaplan‐Meier analysis, we introduced a definition of the event when a patient was deemed to have dropped out. Our definition required the completion of a single ePRO within three months of the end of data cut‐off. This allowed all patients to have an equal and minimum period to engage and was chosen pragmatically because we felt that a three month reporting frequency to be the minimum clinically valuable threshold. However, with a longer gap between study end and last reported ePRO, the Kaplan‐Meier curve would have demonstrated a different level of engagement. Our Kaplan‐Meier analysis shows that patients’ engagement tends to decrease over time, with a median engagement rate of around 15 months. Although imperfect, the analysis does highlight the need for further research to understand patient reasons for dropping out to maximize longer‐term engagement, especially if remote ePRO reporting is to be used more broadly in clinical practice. It also highlights how promising engagement rates in the first six months of use cannot reliably be extrapolated over the longer term.

To provide other perspectives on app use, we examined the proportion of patients enrolled who submitted at least one RAPID3 report per month. We identified a reduction from 91% at baseline to 24% at month 12. However, it is important to note that the patients who monthly completed ePROs were not necessarily the same each month. This again highlights the limitations of existing metrics for user engagement, and alternative approaches are needed to gain a full picture of long‐term usage patterns.

These data confirm a substantial participant disengagement over time. This remains a fundamental challenge for the real‐world adoption of remote monitoring using ePROs.^10^ Our findings match with those from a study by Crouthamel et al,^30^ who reported high initial patient engagement with a smartphone app followed by high attrition with 40.6% of patients completing at least one study assessment at week 2, to only 11.3% at week 12. Nowell et al^33^ reported a similar level of attrition with dropout rates of 22.3% at month 1, followed by 7.9% and 13.8% at months 2 and 3, respectively.^33^ Analysis of National Early Inflammatory Arthritis Audit PROs data identified similar levels of disengagement with only 35% of ePROs completed at baseline,7% at month 3, and around 3% at month 12.^34^

Some research studies have demonstrated higher retention rates for ePROs apps.^11^, ^12^, ^16^, ^35^ However, most of these studies were short‐term (<12 months), with intensive input from the research team. Furthermore, by the nature of agreeing to participate in a research study, the participants are more likely to be an engaged and motivated group of patients. Although unavoidable in any research study, this doesn't reflect routine clinical practice and limits the extrapolation of findings into the general population.

Despite reasonably high attrition, our analysis didn't identify any differences between follow‐up and lost to follow‐up user subpopulations when considering gender, age, IA type, and baseline arthritis severity. This finding was reassuring and suggests that patients lost to follow‐up were not overtly different from those in the follow‐up group. However, we were unable to account for all possible variables, so there may be other unmeasured factors that influence this outcome.

Tobit regression models were used to examine associations of demographics, and baseline disease‐related parameters with such outcomes as elapsed days, number of ePROs, and days between ePROs in the follow‐up group. In our cohort, older patients with IA (aged 60–69) were more likely to be long‐term users of the app than those aged younger than 50 years (*P* < 0.006), and patients aged over 60 years provided more reports than younger users (*P* < 0.0001). This finding aligns with prior research that showed that older age is significantly associated with increased engagement and retention.^21^, ^35^ These results challenge the common assumption that digital participation is more predominant among younger patients, highlighting that digital tools are acceptable by older patients. It also suggests that digital engagement of younger users cannot be taken for granted, and future work should focus on its barriers and drivers. Our results also show that there does not appear to be any gender association with engagement and retention and are aligned with previously reported findings.^21^

As with many digital tools, we found that cumulative enrollment and monthly engagement of patients with IA with the app rose markedly following April 2020, reflecting the impact of the COVID‐19 pandemic on clinician and patient behavior and their interaction with technology and devices.^36^ Although uptake rates have plateaued, this change in behavior has opened the door to wider integration of remotely captured ePROs into clinical settings. This could enable the adoption of innovative patient pathways to facilitate remote triage and monitor disease activity fluctuations accurately and at scale.^37^ From a patient's perspective, this can result in fewer site visits, reducing the burden of travel and the time commitment of hospital appointments.^38^

A surprising finding from this research is that more severe or disabling IA symptoms at baseline do not impact the level of a patient's long‐term engagement or retention rate with the studied app‐based diary. This challenges a commonly held assumption that patients are more likely to engage with digital tools when they have a chronic condition^39^ and feel unwell.^40^ This can provide some reassurance that remote ePRO monitoring does not appear to be biased toward reflecting negative patient experiences related to baseline disease activity.

This is the first analysis demonstrating real‐world engagement and retention in using an ePRO app among patients with IA over a timespan of four years. Although we offer insights into the real‐world integration of digital health tools, the results of our analysis should be interpreted within the context of certain limitations. Firstly, we have not been able to account for all participants’ demographic and socioeconomic characteristics. Details including ethnicity, employment status, years of education, and income level were not available and may impact patient engagement and retention.^34^ This is of importance because health inequality exists for underserved populations, which likely extends to access to digital tools. Without data on social deprivation, it is impossible to fully assess the efficacy of digital interventions across all sections of society.^41^, ^42^ Unfortunately, these data were not available in our cohort with IA.

The duration of symptoms and concomitant disease‐modifying treatments were not captured within our baseline dataset, so we cannot comment on how this might be associated with subsequent patterns of app engagement. Nonetheless, our data was obtained in a real‐world clinical setting and was available to all patients with IA and the required technology. Further research is required to examine those patients lost to follow‐up to explore the reasons for nonengagement. Additionally, more active involvement of health care professionals in encouraging patients to use monitoring tools and discussing the benefits could reinforce engagement. Novel digital personalized notifications could also encourage app use.

High participation attrition in digital research studies reduces study power. Although we identified a high attrition rate in app use over the medium to longer term, our study can help researchers plan similar digital research to make accurate sample size calculations. These data can also help clinical service providers with realistic expectations of app use when implementing remote ePRO monitoring in a routine clinical environment. This research highlights that more work is needed to understand barriers to remote monitoring using ePROs, particularly in the longer term, especially if clinical services are to be built around them.

Our analysis of real‐world data demonstrates the feasibility of using smartphone apps to remotely capture ePROs from patients with IA at scale and over a long term. Despite the continued success of digital health tools in research, several barriers hinder participants’ long‐term engagement and retention. This analysis offers insights into what might be expected regarding engagement and the long‐term sustainability of remote monitoring using an app to capture ePROs in routine clinical settings. Understanding real‐world performance, factors, and strategies will allow clinicians and researchers to focus on areas that can improve accessibility, inclusion, user retention and reduce dropout rates. This is critical to realizing the promise of remote monitoring to improve clinical services and facilitate the development and implementation of innovative strategies to provide high‐quality health care.

## AUTHOR CONTRIBUTIONS

All authors contributed to at least one of the following manuscript preparation roles: conceptualization AND/OR methodology, software, investigation, formal analysis, data curation, visualization, and validation AND drafting or reviewing/editing the final draft. As corresponding author, Dr Hamann confirms that all authors have provided the final approval of the version to be published and takes responsibility for the affirmations regarding article submission (eg, not under consideration by another journal), the integrity of the data presented, and the statements regarding compliance with institutional review board/Declaration of Helsinki requirements.

## Supporting information


**Disclosure form**.

**Supplementary Figure S1:** App screen shots: patient (a, b) and clinician (c) user interfaces.
**Supplementary Figure S2:** Cumulative patient downloads (a) to use app and (b) pattern of first baseline ePRO completion
