# Supplementary material for: How and Who? Examining Real‐World Evidence of Engagement and Use of an Electronic Patient‐Reported Outcome Smartphone Application in Routine Clinical Care for Patients With Inflammatory Arthritides
Source: ACR Open Rheumatol. 2025 May 6;7(5):e70049. doi: 10.1002/acr2.70049 (PMC12053926; doi:10.1002/acr2.70049)
Supplement: Supplementary file 2 — Supplementary Figure S1: App screen shots: patient (a, b) and clinician (c) user interfaces. Supplementary Figure S2: Cumulative patient downloads (a) to use app and (b) pattern of first baseline ePRO completion [file ACR2-7-e70049-s001.pdf]

Supplementary Figure S1. App screen shots: patient (a, b) and clinician (c) user interfaces

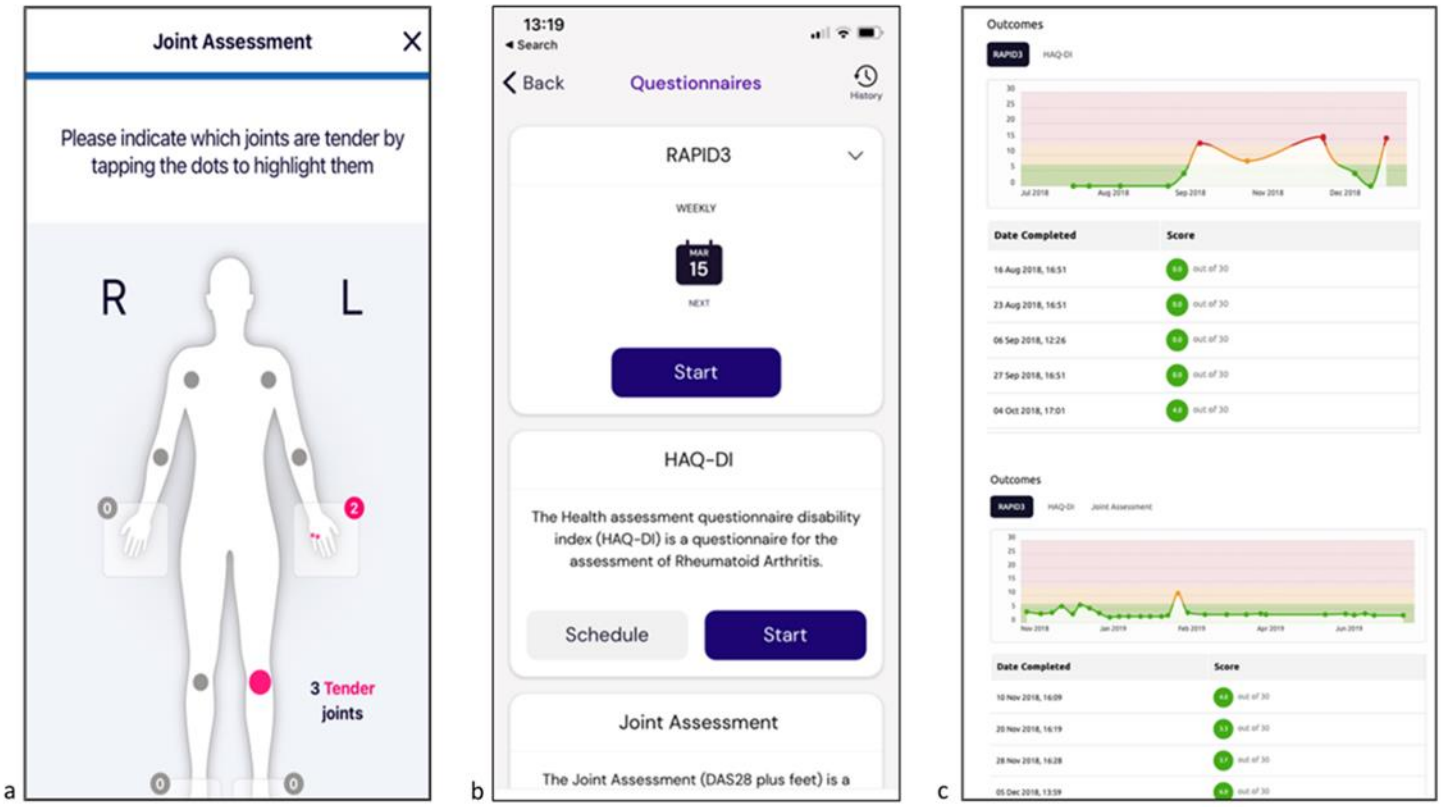

Supplementary Figure S2. Cumulative patient downloads (a) to use app and (b) pattern of first baseline ePRO completion

a.

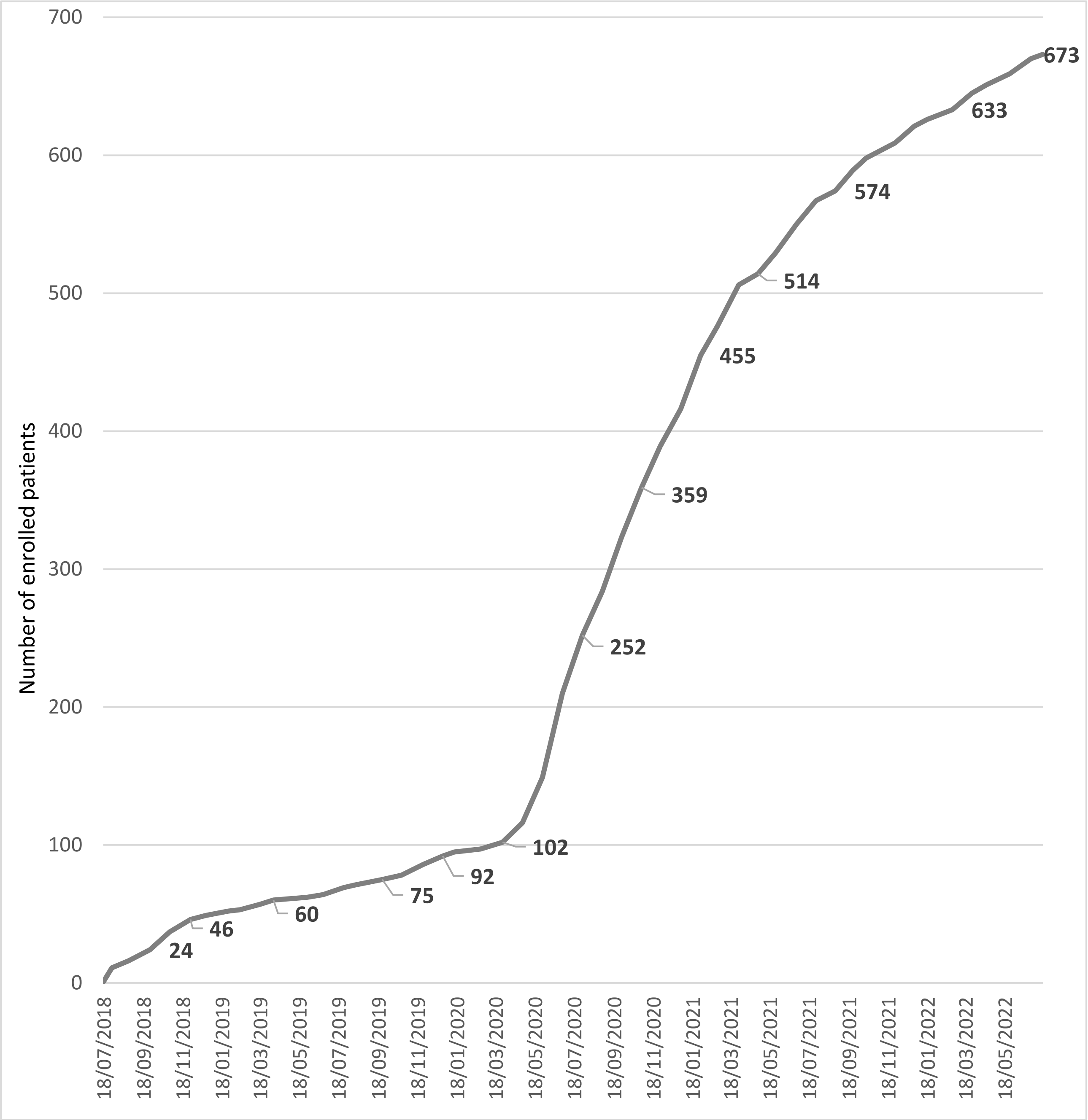

b.

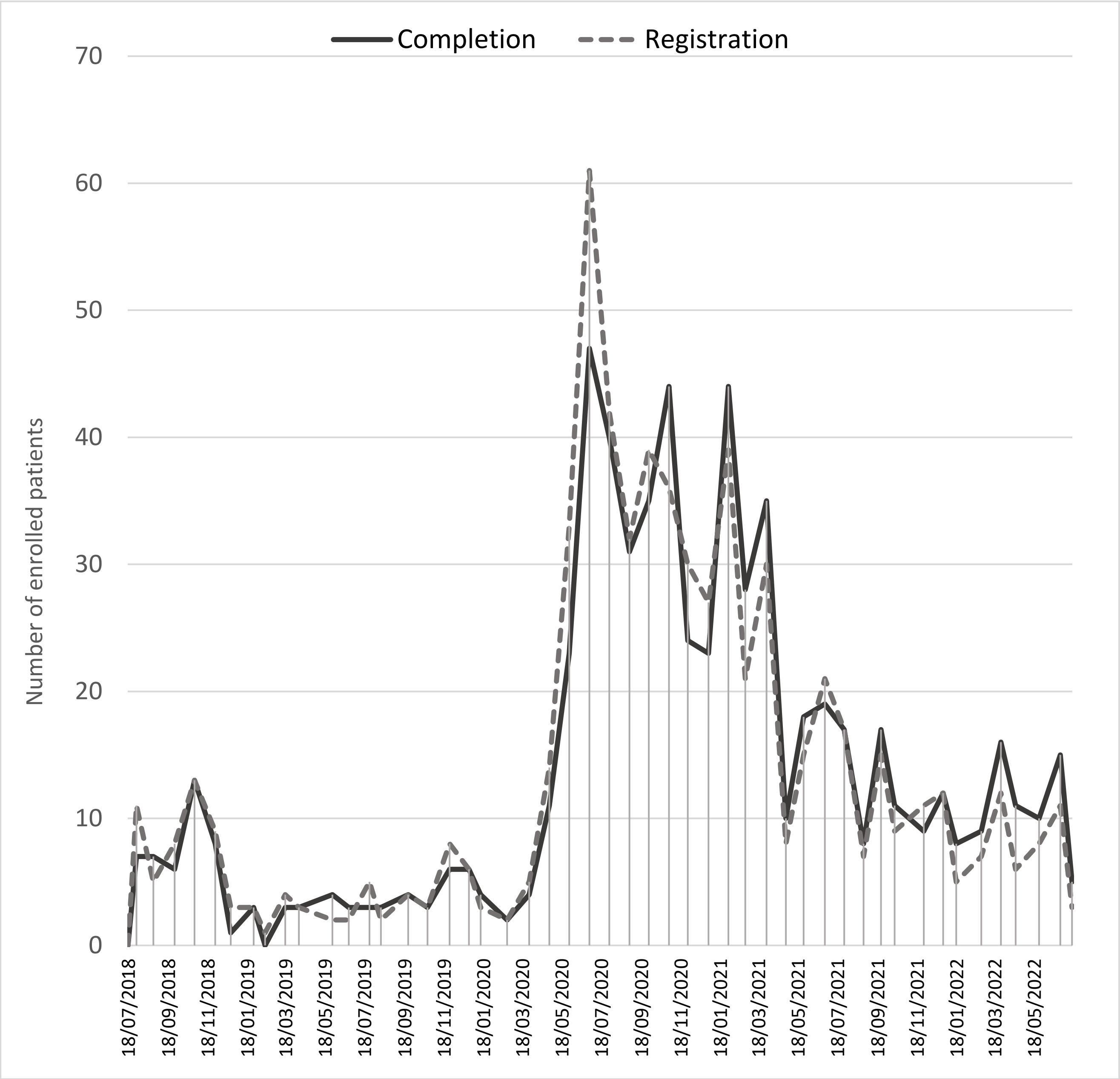

ePRO: electronic patient-reported outcome
